# Comparison of the Joint Space in Different Types of Malocclusion Using Three-Dimensional Models

**DOI:** 10.18502/ijph.v49i7.3596

**Published:** 2020-07

**Authors:** Eun-Young JEON, Jeong-Hyun LEE, Jong-Tae PARK

**Affiliations:** 1. Department of Dental Hygiene, Kyung Bok University, Namyangju, South Korea; 2. Department of Oral Anatomy, College of Dentistry, Dankook University, Cheonan, South Korea

## Dear Editor-in-Chief

Harmonious functioning of the temporomandibular joint (TMJ) is crucial to the correct operation of the masticatory system, which makes ideal positioning of the condyle in the TMJ clinically important (1). The joint space is determined by the size of the glenoid fossa and condyle and the position of the condyle within the glenoid fossa (2), and so the joint space is used as a marker to assess condylar positioning in the glenoid fossa (3). Many studies have analyzed the joint space in patients with malocclusion (4–5). 2D techniques cannot accurately measure the narrow 3D joint space. Therefore, 3D models are more appropriate for joint-space measurements (6).

This study was approved by the Institutional Review Board of Dankook University Dental Hospital (DUDH IRB 2015-12-022).

**Fig. 1:**
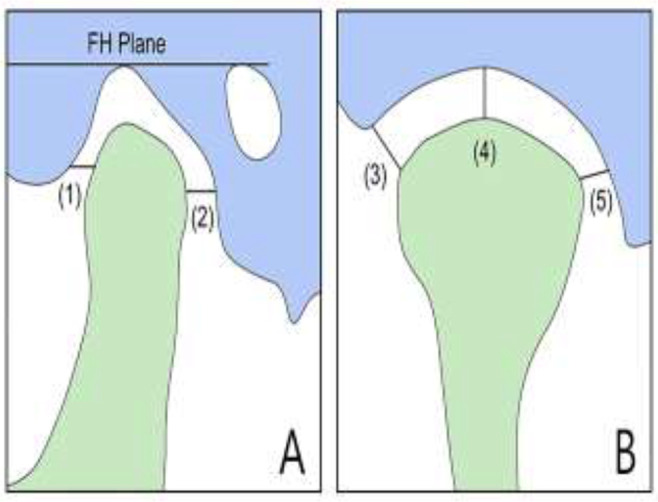
Measurements of joint space (A. sagittal view, B. coronal view, (1). AJS, (2). PJS, (3). LJS, (4). SJS, (5). MJS)

**Fig. 2:**
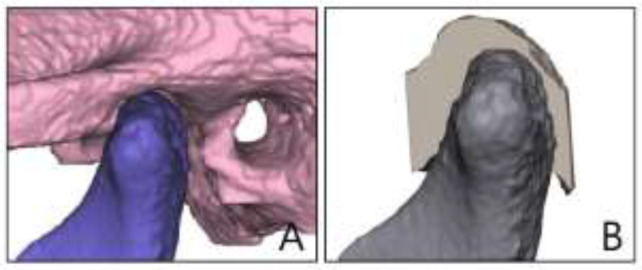
3D models of patients with malocclusion (A. reconstruct from mimics program, B. reconstruct from freeview program)

CBCT data were obtained for 60 patients admitted to the Orthodontics Divisions in the Department of Oral and Maxillofacial Radiology at Dankook University. The CBCT data of 60 patients with malocclusion were obtained in the Digital Imaging and Communications in Medicine (DICOM) format from a CBCT scanner (Alphard 3030, Asahi, Kyoto, Japan). The DICOM files were imported into Mimics software (Materialise, Leuven, Belgium) for constructing 3D models of the skull. Since the joint space corresponds to an empty space, the Mimics files of the 3D-reconstructed skulls were imported into Freeform software (3D Systems, Rock Hill, SC, USA) to allow accurate measurements to be made. Areas corresponding to the condyle, fossa, and joint space were cropped out, and the area corresponding to the joint space was filled to construct a 3D model. We reconstructed the CBCT data of patients with malocclusion of classes I, II, and III as 3D models and measured the joint space at different locations to facilitate comparisons of the spatial properties of the TMJ Figs. 1, 2.

A Kruskal-Wallis test was used to analyze the joint space according to different types of malocclusion. Significant differences in the measured left and right joint-space values were found between the three experimental groups: in the anterior joint space (AJS) (χ2=12.473 and *P*=0.002 on the left, and χ2=7.868 and *P*=0.020 on the right), superior joint space (SJS) (χ2=18.565 and *P*<0.001, and χ2=13.937 and *P*=0.001, respectively), and lateral joint space (LJS) (χ2=8.237 and *P*=0.016, and χ2=9.463 and *P*=0.009, respectively). A significant interclass difference was observed for the left medial joint space (MJS) (χ2=11.878, *P*=0.003). A post-hoc Mann-Whitney U test adjusted using the Bonferroni correction method revealed significant differences in the left and right AJS, SJS, LJS, and left MJS measurements (Table 1).

**Table 1:** Average rank and result of kruskal wallis test of joint space measurements of subjects in three subgroup

*** Measurements ***		*** Mean number ***	*** Chi-square ***	*** P-value ***	*** B.C. method ***
LAJS	ClassI	19.60	12.473	0.002^*^	ClassI <classII, III
	ClassII	33.50			
	ClassIII	38.40			
LPJS	ClassI	32.10	1.637	0.441	-
	ClassII	32.95			
	ClassIII	26.45			
LSJS	ClassI	31.35	18.565	0.000^**^	ClassIII<classI, classII
	ClassII	41.95			
	ClassIII	18.20			
LLJS	ClassI	30.45	8.237	0.016^*^	ClassIII<classII
	ClassII	38.45			
	ClassIII	22.60			
LMJS	ClassI	29.20	11.878	0.003^*^	ClassIII<classII
	ClassII	40.60			
	ClassIII	21.70			
RAJS	ClassI	22.45	7.868	0.020^*^	ClassI <classII
	ClassII	37.90			
	ClassIII	31.15			
RPJS	ClassI	25.40	2.591	0.274	-
	ClassII	33.55			
	ClassIII	32.55			
RSJS	ClassI	36.20	13.937	0.001^**^	ClassIII<classI, classII
	ClassII	36.70			
	ClassIII	18.60			
RLJS	ClassI	29.50	9.463	0.009^*^	ClassIII<classII
	ClassII	39.45			
	ClassIII	22.55			
RMJS	ClassI	34.25	2.004	0.367	-
	ClassII	26.45			
	ClassIII	30.80			

* *P*-value were obtained by Kruskal-Wallis(* P*<0.05)

** *P*-value were obtained by Kruskal-Wallis(p<0.001)

The same characters were not significant by bonferroni correction method(B.C. Method)/ .05/3=0.167

The joint space is constituted empty space using 3D models (6). However, measuring the joint space between the glenoid fossa and condyle using 3D software requires a connection between the outermost edge of the condyle, used as a reference point, and the innermost edge of the glenoid fossa. The PJS and AJS must be measured parallel to the Frankfort plane, and considerable interexaminer errors arise during this process. To overcome these limitations, the present study filled the joint space using Freeform software before measuring it. We found that the measured joint space varied with the type of malocclusion. The obtained results will provide deeper insights into malocclusion and TMJ shapes, and suggest that malocclusion can contribute to TMJ deformation.
